# The effect of ocean alkalinity enhancement on pelagic bacterial communities: focus points derived from a mesocosm experiment

**DOI:** 10.3389/frmbi.2025.1606890

**Published:** 2025-08-11

**Authors:** Dominik Antoni, Antje Wichels, Maarten Boersma, Gunnar Gerdts

**Affiliations:** ^1^ Alfred-Wegener-Institut Helmholtz-Zentrum für Polar- und Meeresforschung, Biologische Anstalt Helgoland, Helgoland, Germany; ^2^ Alfred-Wegener-Institut Helmholtz-Zentrum für Polar- und Meeresforschung, Wadden Sea Station Sylt, List, Germany; ^3^ Faculty 02: Biology/Chemistry, University of Bremen, Bremen, Germany

**Keywords:** ocean alkalinity enhancement, negative emission technology, environmental microbiology, marine microbiology, carbon sequestration, mesocosm experiment, amplicon sequencing, bacterial cell counts

## Abstract

Anthropogenic climate change caused by CO_2_ emissions forces humanity to reduce the usage of fossil fuels. Along with the task of emission reduction, societies face the task of removing excess CO_2_ from the atmosphere by using negative emission technologies (NETs). Ocean alkalinity enhancement (OAE) is a proposed NET, aiming at increasing oceanic CO_2_ uptake through the addition of alkaline substances. This is an anthropogenically accelerated version of rock weathering, a natural global process for atmospheric CO_2_ regulation. The environmental impacts of OAE remain poorly understood. This study was part of a comprehensive OAE-mesocosm experiment in the North Sea (RETAKE), and focused on the effects of OAE on the pelagic bacterial community during the experiment. We assessed changes in bacterial community structure with 16S rRNA amplicon sequencing and abundance with flow cytometry, to evaluate responses to alkalinity addition. Beta diversity analysis showed that sampling time was the primary driver for community variation, with only marginal structural differences linked to alkalinity treatments. PERMANOVA tests conducted on predictions of functional metabolic pathways of the community revealed significant differences between treatments and baseline controls. A deeper analysis of the identified metabolic pathways revealed little evidence for alkalinity-induced changes. In contrast, total bacterial cell counts were influenced by alkalinity additions, showing delayed abundance peaks at higher concentrations and a non-linear response threshold between 500–750 µmol/L. These dynamics were linked to shifts in chlorophyll concentrations, suggesting an indirect effect of OAE on bacteria mediated by phytoplankton derived resources. This study is one of the first to assess ecological impacts of OAE on bacteria. Our findings highlight a structural resilience of bacterial communities to OAE but also show a quantitative response. By discussing our findings, this study aims to provide focus points, such as a threshold for save levels of alkalinity addition, to direct future research.

## 1 Introduction

The industrial revolution in the 18th century marked the beginning of large-scale use of fossil fuels for energy accompanied by a substantial increase in CO_2_ emissions. Since preindustrial times, the total amount of CO_2_ in the atmosphere has increased from 280 ppm to 420 ppm in the year 2022 (Hashimoto and Hashimoto, 2019; Friedlingstein et al., 2024). Elevated levels of CO_2_ in the atmosphere are contributing to global warming and climate change, resulting in an increase of approximately 1°C in global average temperature since preindustrial times (Pörtner et al., 2022). The rising atmospheric CO_2_ concentration is enhancing its diffusion into the ocean, reacting with water to create carbonic acid, thereby releasing hydrogen ions. This process is called Ocean Acidification and since preindustrial times the relative abundance of hydrogen ions in the seawater has increased by 30% (Raven et al., 2005). Even under the most favorable scenario developed by the intergovernmental panel on climate change (IPCC), CO_2_ emissions will need to be brought down considerably. Hence, in 2015, a total of 190 countries agreed to mitigate global warming by adopting the 1.5°C and 2°C climate goals in the Paris agreement. The main objective is to restrict global warming to 1.5°C and 2°C, respectively, by limiting CO_2_ emissions (Rhodes, 2016).

With the goals of reaching net zero emission during the 21^st^ century, there is a need for carbon dioxide removal (CDR) strategies, to compensate for emissions that are difficult to mitigate, such as those from air traffic. Negative emission technologies (NETs) are anthropogenic practices that remove more CO_2_ from the atmosphere than they emit. The AR5 report of the IPCC evaluated multiple model scenarios for their likelihood of meeting the 2°C climate goal. The vast majority (87%) of these promising scenarios incorporated CDR in the latter half of the 21^st^ century. These modelling results emphasize the crucial role of NETs in the future (Smith et al., 2016).

One example of such a NET is ocean alkalinity enhancement (OAE). Alkalinity is the buffering capacity to resist pH changes upon acidification. Chemically, alkalinity is defined as the sum of all proton acceptor molecules minus all proton donor molecules in the solution (Montserrat et al., 2017). Ocean alkalinity can be increased by adding alkalizing substances like sodium hydroxide into the water (Hartmann et al., 2022). Other methods of alkalinity enhancement can involve adding particles of quick weathering rocks, such as olivine (Schuiling and De Boer, 2010) and calcite (Caserini et al., 2022), to seawater. As a NET, OAE removes CO_2_ from the atmosphere and stores it in the ocean by shifting the carbon equilibrium from gaseous CO_2_ towards dissolved bicarbonate (HCO_3_^-^) and carbonate (CO_3_^2-^). The resulting depletion of CO_2_ in the water enhances its diffusion into the water, ultimately leading to a reduction of atmospheric CO_2_ levels (Vicca et al., 2022). In addition, the elevated alkalinity helps to counteract ocean acidification by enhancing the oceans buffering capacity (Renforth and Henderson, 2017; Harms et al., 2024).The concept of OAE as a CDR strategy is still relatively new, with limited research available into potential environmental risks. Hypotheses concerning the effects of OAE on the environment are predominantly positive. The main reason for that is the presumed potential to counteract the adverse effects of ocean acidification, on organisms such as calcifying coccolithophores (D’Amario et al., 2020) and sponges (Kleypas et al., 1999; Figuerola et al., 2021). The current literature available on the ecotoxicological effects of OAE is largely theoretical and potential effects are only predicted based on the chemical changes caused by it. One important aspect influencing the anticipated chemical changes from OAE is the substance used for alkalization. In a comprehensive study on environmental risks and co-benefits of OAE, the authors propose that different materials used are likely to benefit different marine organisms (Bach et al., 2019). OAE using weathering products from calcite rocks is likely to benefit calcifying organisms like coccolithophores, while silicate rock weathering products, such as from olivine, may support silicifying organisms like diatoms. The authors refer to this as the ‘white or green ocean hypothesis’, as enhanced growth of the respective algae is associated with the different color, white for calcifers and green for silicifiers. Aside from the material used for OAE the equilibration status of the application is also considered as an important aspect in its ecotoxicology. Generally, a distinction is made between CO_2_ equilibrated and unequilibrated OAE. Alkalization increases the pH, which reduces the availability of dissolved CO_2_ and bicarbonate in the seawater (Wolf-Gladrow et al., 2007). Equilibrated OAE refers to an alkalinity increase without requiring extended ingassing of CO_2_ from the atmosphere to equilibrate dissolved inorganic carbon concentrations again. This constitutes a milder stressor compared to unequilibrated OAE, which induces a sudden pH spike upon abrupt alkalinity addition (Suessle et al., 2025). Large-scale application of equilibrated OAE involves the use of weathering- or bioreactors to evenly add dissolved alkalinity into the environment (Ferderer et al., 2022; Hartmann et al., 2022; Hutchins et al., 2023). This represents the construction of additional infrastructure, making equilibrate OAE scenarios more costly compared to unequilibrated OAE (Gattuso et al., 2021). The effects of OAE on the marine ecosystem remain uncertain, presenting a critical bottleneck for its application.

The ocean plays a fundamental role in carbon sequestration, serving as a sink for ~ 2.8 gigatons of carbon annually (Friedlingstein et al., 2023). Furthermore, the ocean contributes equally to the global primary production as terrestrial ecosystem (Field et al., 1998; Moran et al., 2022). Marine bacteria are essential in the ecosystem as they have a short generation time and high turnover rates in the recycling of nutrients (Azam and Malfatti, 2007). Certain bacterial taxa form mutualistic relationships with diatoms, in which they fix nitrogen and supply phosphorus and other nutrients that support diatom growth (Amin et al., 2012). In a trophic pathway known as the microbial loop bacteria remineralize carbon and other nutrients to make them available again for primary production in the surface waters (Heinrichs et al., 2020). Marine snow refers to a phenomenon where organic material from algal primary production aggregates into sinking particles that are colonized by bacteria. These particles act as nutrient hotspots in an otherwise nutrient-poor water column and are recycled by heterotrophic bacteria, making the nutrients available again to the surrounding water (Borer et al., 2023). In addition, marine snow serves as a carbon sink because these particles can sink to the deep ocean, where they are stored for millennia, with the associated bacterial community turning over as the particles descend (Mestre et al., 2018).

Modern advances in next-generation sequencing (NGS) technologies allow for a qualitative description of entire bacterial communities. These NGS methods have increasingly been applied to marine environments to investigate changes in bacterial communities caused by pollutants such as microplastics (Song et al., 2022) or wastewater discharge (Kodera et al., 2023). In addition, NGS methods are increasingly subject of monitoring programs for aquatic environments (Paruch and García-Aljaro, 2024). Large-scale surveys such as the Tara Ocean sampling campaign have mapped reoccurring bacterial community patterns in the oceans, thereby advancing the understanding of the ecological significance of bacterial communities across diverse marine habitats (Sunagawa et al., 2015). Despite the wealth of information on bacterial communities in natural environments, only one study has investigated the impact of OAE on seawater bacterial communities using NGS methods (Ren et al., 2021). In that study, the authors implemented olivine in an OAE experiment to examine shifts in both free-living and particle-associated bacterial communities and found that olivine-based OAE primarily affected particle-associated bacteria. Distinguishing between particle-attached and free-living bacterial communities is reasonable in an experiment, given their different ecological roles in the ocean. Particle-attached bacteria are focused on degrading matter, while free-living ones are focused on locating and absorbing dissolved nutrients (D’ambrosio et al., 2014). This division may, however, be premature as empirical studies on the effects of unequilibrated OAE on bacterial communities have not been conducted in general. This and the investigations into a safe threshold for alkalinity addition are critical research gaps in the ecotoxicology of OAE.

In this study, we analyzed the effects of OAE on the bacterial community during a large mesocosm experiment in the North Sea at the south harbor of Helgoland. The experiment was conducted by the RETAKE consortium under the CDRmare research mission and was funded by the German Ministry of Education and Science through the German Alliance for Marine Research (DAM) “https://retake.cdrmare.de”. The study design specifically aimed to investigate the effects of OAE treatments on the coastal marine environment without equilibrating the water with atmospheric CO_2_, while applying a gradual range of alkalinity additions. This is to our knowledge the first major study to have analyzed a wide range of alkalinity addition levels and their effects on bacterial communities. We hypothesize that alkalinity-driven changes will be observable in the beta diversity of the bacterial community samples taken during the experiment. We expect to see shifts in the community composition that can be linked to increased alkalinity gradients in the experiment. The treatments may affect carbon metabolism, as unequilibrated OAE reduces availability of dissolved inorganic carbon. Accordingly, we expect to observe increases in the abundance of predicted functional pathways associated with carbon processing within the bacterial community of treated mesocosms. The resulting reduction of CO_2_ availability also leads us to expect altered primary production in mesocosms with increased alkalinity treatments. This is likely to impact heterotrophic bacterial growth. We hypothesize that the cumulative bacterial cell counts will decrease in mesocosms receiving higher concentrations of alkalinity.

The insights generated from this study provide a foundation for future research by identifying key areas for further investigation, such as a safe threshold for alkalinity addition and interactions between bacteria and primary producers during OAE. This represents an initial step towards establishing a knowledge base for defining a safe operating space for OAE and assessing whether its potential environmental impacts are acceptable in view of its projected benefits for CO_2_ drawdown.

## 2 Materials and methods

### 2.1 Study site and treatments

Twelve mesocosms were deployed in the southern harbor of Helgoland from March 12, 2023, to April 21, 2023 (Dummermuth et al., 2023). The timeframe was specifically chosen to capture the phytoplankton spring bloom. The mesocosms were constructed from flexible plastic bags strapped to floating frames, designed to hold 7800 L of water while floating in a natural marine environment. This setup has been used in previous mesocosm studies on OAE (Marín-Samper et al., 2023) and ocean acidification (Spilling et al., 2016; Langer et al., 2017). For more detailed descriptions of the mesocosms, see Riebesell et al. (2013) and Goldenberg et al. (2022). After deployment, the mesocosms were initially filled with seawater from the Helgoland Roads observatory site (Wiltshire et al., 2010). The water was filtered with a <3 mm mesh sized net upon filling of the mesocosms.

The experimental setup included 12 mesocosms, divided into two groups of six. The experiment was designed to test different alkalinity dilution scenarios, where immediately diluted alkalinity is added to the entire mesocosm, in contrast to delayed diluted alkalinity with high localized concentrations within a top layer of a mesocosm. The dilution scenarios were invented to test effect of point-based perturbation of high alkalinity against uniform dissolution over a larger area. One group assessed the impacts of immediately even diluted alkalinity and is called the IMM group, while the other assessed a delayed dilution of alkalinity, called the DEL group.

The perturbation simulated calcium mineral-based OAE using sodium hydroxide (NaOH) under non-equilibrated atmospheric conditions. To mimic the presence of dissolved calcium minerals, a stock solution of calcium chloride (CaCl) was first prepared using 17 L of pre-filtered freshwater. This solution was introduced into the mesocosms using a horizontally-distributing device called the “spider” (Riebesell et al., 2013). An additional 17 L of freshwater was subsequently flushed through the spider to ensure complete delivery of the stock solution and to aid in establishing a distinct vertical salinity gradient (ΔS ≈ 0.5 PSU), thereby creating two layers in the DEL mesocosm. After the stratification was established, the NaOH stock solutions, also prepared with 17 L of pre-filtered freshwater, were added in the same manner using the spider device. The spider device ensures even horizontal distribution throughout the mesocosm water along the depth. The water had a background alkalinity of 2330 µmol/L. Alkalinity was added in six steps of 250 µmol/L, ranging from Δ0 µmol/L as the control mesocosms to Δ1250 µmol/L as the highest alkalinity concentration (scheme in supplement). The alkalinity addition and the pH were monitored over the course of the experiment in each mesocosm via titration of 20 ml of mesocosm water (supplement).

### 2.2 Sampling

Water samples were taken from the mesocosms using a 2.5 meter long tube sampler with 5 liter capacity (Marín-Samper et al., 2023). The sampler had valves at both ends and was inserted into the mesocosms with open valves. The valves were sealed upon withdrawal to ensure a depth integrated sample. Samples of 15–20 L were collected every second day and transported in multiple canisters to the laboratory for further analysis. During the layering period, samples were not taken with the tube sample, but with a Niskin bottle to keep the integrity of the layering in the bottom and top samples. The layering period lasted for three days.

Biomass samples for amplicon sequencing of the bacterial community were taken weekly with either 6 or 8 days apart from each other, alternating between Wednesdays and Thursdays. This shift had to be implemented due to the samplings of the campaign being every second day. The biomass from 2 L of water was taken by filtering it on 0.22 µm Sterivex cartridge filters (Merck, Darmstadt, Germany, Cat No: SVGP01050) using a peristaltic pump equipped with six tube channels to simultaneously process multiple samples (DeHart et al., 2023). The filters were then stored at -80°C for downstream analysis after the remaining water was blown out using a syringe with a sterile filter.

For bacterial cell counts, 2 mL of water was collected from each mesocosm in cryovials every second day. Formaldehyde (final concentration 1%) was added to each cryovial, and the sample was incubated at 4°C for 24 hours for cell fixation. After incubation, they were stored at -80°C until further processing.

### 2.3 DNA extraction and amplicon sequencing

DNA extraction from the Sterivex filters was performed by opening the capsules with a clean pipe cutter and retrieving the filter units (Cruaud et al., 2017). Each filter unit was placed into a Bead Pro Tube from the DNeasy PowerWater kit (Qiagen, Hilden, Germany). DNA extraction followed the manufacturer’s instructions with the following modifications to optimize yield: After adding the solution PW1 from the kit (step 5), the Bead Pro Tube was incubated at 60°C for 10 minutes to lyse organisms with robust cell walls. The tubes were then vortexed and centrifuged at 1500 g for one minute to separate the DNA from the filter. Following filter removal, an additional centrifugation step for 1 min at 1500 g was performed and the DNA extraction was continued at step 8 according to the manufacturers guide.

DNA quantification was conducted using a NanoQuant plate reader (Tecan Group Ltd., Männedorf, Switzerland) (Bruijns et al., 2022). From each sample 1 ng/µL of DNA was sent to LGC (Berlin, Germany) for library preparation and sequencing. A brief description of the PCR and sequencing procedure: The V4-V5 regions of the 16S rRNA genes were amplified with PCR (see supplement for the used chemicals and cycling conditions) with universal primers 515F-Y and 926R (Parada et al., 2016). Amplicons were purified using Agencourt AMPure XP beads (Beckman Coulter, Inc., IN, USA) and MiniElute columns (QIAGEN GmbH, Hilden, Germany) to remove non-specific products before library construction. The samples were dual indexed with the Ovation Rapid DR Multiplex System (NuGEN Technologies, Inc., CA, USA). Sequencing was performed on an Illumina MiSeq platform with V3 Chemistry, generating 5 million read pairs. The raw sequencing data is uploaded at the NCBI with the accession number: PRJNA1245293

### 2.4 Bioinformatical processing and ASV-generation

Read processing was conducted in RStudio using the *DADA2* package (Version 1.36.0) (Callahan et al., 2016). Demultiplexed, merged reads with removed primers underwent quality control, filtering for base positions with an average Phredscore of 30 (Prodan et al., 2020). The reads were filtered to have a minimum length of 170 and a maximum length of 350 base pairs. Dereplicated reads were processed with the dada() function, generating Amplicon Sequence Variants (ASVs) (Eren et al., 2013) using a run-specific error model. Post-inference, chimeras were removed, and taxonomic classification was performed using the IdTaxa() function (Murali et al., 2018) in the *DECIPHER* package (Version 3.4.0) (Wright, 2016), utilizing the SILVA 138 SSU database (release date 16.12.2019) (Quast et al., 2012). Non-bacterial ASVs were excluded from the analysis. The code used for the ASV generation is provided at a GitHub repository: https://github.com/Dom-Antoni/RETAKE_Analysis/tree/RETAKE-Microbiome

### 2.5 Bacterial community analysis

To assess the overall structure of the microbial community data, we conducted Principal Coordinates Analysis (PCoA) based on a Bray–Curtis dissimilarity matrix with the *phyloseq* package (McMurdie and Holmes, 2013) and the *vegan* package in R (Oksanen et al., 2013). Square root transformed count data, rarefied to the lowest sequencing depth of 5361 ASVs was used (Schloss, 2024).

The Bray–Curtis metric was chosen because it incorporates relative taxonomic abundances, which are central to our analysis, as shifts in abundance are considered key indicators of ecological change (Bray and Curtis, 1957). We opted against using phylogenetic-based metrics such as UniFrac, as these emphasize evolutionary relationships, which happen in mesocosms regardless of alkalinity treatments. PCoA was applied to the entire dataset, and the ordinations were visualized with facets for alkalinity treatment concentrations and sampling time points to evaluate how each parameter aligns along the dominant axes of variation.

To further examine whether patterns in community shifts differ between ecologically distinct subgroups, we repeated the PCoA analysis with subsets of the bacterial community representative of the core and transient members of the microbiomes. Core Amplicon Sequence Variants (ASVs) were defined as those present in at least 50% of all samples, resulting in 139 ASVs representing 26,765 sequence counts. Transient ASVs included all remaining taxa, comprising 2,169 ASVs with a total of 30,695 counts.

To identify patterns in the taxonomic microbial community composition beyond predefined experimental groupings, we applied an unsupervised clustering approach based on community similarity. Unsupervised means that prior sample groupings based on metadata of the sample origin, such as alkalinity addition and sampling day, were ignored, and an algorithm was used to find patterns in the data. This strategy was necessary because the effects of alkalinity treatment levels and sampling time points are not distinguishable from each other in the applied study design, making it difficult to attribute observed community changes to a single factor.

This unsupervised clustering included the K-means clustering algorithm (Lloyd, 1982) to group samples with similar bacterial communities together, allowing for the identification of patterns. To determine the optimal number of clusters, we applied Elbow and Silhouette plot analysis (Yuan and Yang, 2019) using the Bray-Curtis dissimilarity matrix. Both analyses indicated that three was the optimal number for clustering (see supplement), which was then used to categorize the samples. After the clustering, we reapplied the sample metadate again, to assess which factors best explain the observed grouping. We performed a chi-square test of independence (Tallarida et al., 1987) on the sampling time point, alkalinity treatment levels and the dilution treatment, as parameters. By comparing the p-values returned from the three tests and identifying the lowest p-value the chi-square test points to the parameter that describes the clustering best.

The primary purpose of the clustering analysis was to visualize patterns in the taxonomic composition of bacterial communities throughout the experiment. Within each of the resulting clusters, relative abundances at the family level were illustrated using stacked bar plots. To identify the ASVs that contributed most to the differences among clusters, a similarity percentage (SIMPER) analysis was conducted using the *vegan* package in R (Clarke, 1993). For visualization, we selected ASVs that cumulatively accounted for up to 70% of the total dissimilarity between clusters, following the threshold recommended by (Clarke and Warwick, 2001), resulting in a subset of 613 ASVs. Alpha diversity of the bacterial community samples was assessed between clusters using richness, Shannon, and Simpson indices calculated with the *vegan* package (Willis, 2019)

### 2.6 Statistical analysis on the bacterial community

To evaluate the effects of alkalinity addition, sampling time, and dilution treatment on bacterial community composition, we conducted Permutational multivariate analysis of variance tests (PERMANOVA) using Bray–Curtis dissimilarity matrices derived from ASV-level abundance data. In contrast to the previous chi-square test on k-means clusters—which examined groups defined by similarity in community composition, this analysis tested for differences between groups explicitly defined by the experimental treatments. This approach aligns with standard practice for assessing treatment effects in experiments.

The PERMANOVAs were performed using the Adonis function in *vegan* to assess the significance of centroids of groupings predefined by alkalinity treatment, the sampling time point and the dilution treatment. Pairwise PERMANOVA comparisons were performed using the pairwise.adonis() function (Martinez Arbizu, 2020) to identify specific group differences as a *post-hoc* test. Bonferroni correction was employed to mitigate Type I errors (Dunn, 1961). Baseline samples taken before treatment application were excluded to avoid a bias on the result from untreated controls.

### 2.7 Predicted functional profiling

To expand the analysis on the bacterial community, we further investigated differences in predicted functional metabolic pathways between treatments. Functional profiles of the bacterial communities samples were predicted with Tax4Fun2 (Wemheuer et al., 2020) a predictive tool that infers functional metabolic profiles of bacterial communities using 16S rRNA gene sequence data. The aim was to test if alkalinity treatment induced shifts in metabolic pathways. Metabolic pathways represent sequences of chemical reactions conducted by cells which are associated with functional mechanisms. These mechanisms are clustered as metabolic pathways with their own taxonomic classification provided by the Kyoto Encyclopedia of Genes and Genomes (KEGG). This is a bioinformatic tool providing databases to interpret genomic data under a more biological relevant context like metabolic functionality (Kanehisa and Goto, 2000).

Alkalinity induced changes in the relative abundance of KEGG pathways identified, were tested by PERMANOVA between samples grouped by alkalinity addition and by sampling time point, with subsequent *post-hoc* test conducted as described above for the taxonomic bacterial community. With a SIMPER analysis we identified KEGG pathways which represent a cumulative difference in the similarity percentage of 70% between baseline samples and treated samples. We focused on these pathways to visualize relative abundance of KEGG pathways with a stacked bar plot.

### 2.8 Chlorophyll *a* concentration measurement

Subsamples (400–1000 ml) were filtered (<200mbar) onto pre-combusted glass fiber filters (25mm diameter, GF/F Whatman, 0.7 µm nominal pore size). Care was taken to minimize exposure to light during the filtration process by covering the filtration racks. Filters were subsequently stored in 2 ml plastic vials at -80°C for 1.5 months until further processing. Pigments were extracted in 100% acetone (Baker 8142, Avantor, Radnor, USA) by homogenizing the filters using 0.5 mm glass beads in a cell mill (Precellys, Montigny-le-Bretonneux, France). Samples were then centrifuged (10 min, 10000 rpm, 4°C), and the supernatant was filtered using a 0.2 µm polytetrafluoroethylene (PTFE) syringe filter (13mm diameter, Lab Logistics Group). The concentration of photosynthetic pigments, including Chlorophyll a in the supernatant was determined through reverse-phase high-performance Liquid Chromatography (Thermo Scientific HPLC Ultimate 3000) following the methodology outlined in Van Heukelem and Thomas (2001).

### 2.9 Total bacterial enumeration with flow cytometry

Flow cytometry was conducted using a FACSCalibur flow cytometer. Bacterial cell counts followed the protocol described by Marie et al. (1999) for bacterioplankton enumeration. Briefly: samples were stained with a 1:10,000 dilution of Sybr Green I stock solution and incubated in the dark at room temperature for 15 minutes. Flow cytometric measurements were recorded using BD CellQuest Pro™ software over a one-minute period. All events were saved in FACS files.

The FACS files were processed using floreader.io, with each file manually gated to isolate the prokaryotic community. The number of events within the gate was divided by the calibrated flow rate (µL/min) to calculate bacterial cell abundance. All gating images and analysis details are available online in the GitHub repository: “https://github.com/Dom-Antoni/RETAKE_Analysis/tree/RETAKE-Microbiome”. Instrument settings and amplification details are provided as well.

### 2.10 Cross-correlation analysis between chlorophyll *a* and bacterial cell counts

To assess the temporal relationship between phytoplankton and bacterial dynamics, we conducted a cross-correlation function (CCF) analysis (Helleseth, 1976) between chlorophyll *a* concentrations (as the independent variable, *x*) and bacterial cell counts (as the dependent variable, *y*) using the ccf() function in R. The CCF calculates correlation coefficients across a range of time lags to identify potential lead-lag relationships between the two variables. In this study, a single time lag corresponds to two days, reflecting the interval between sampling events. The lag at which the maximum correlation occurs indicates the time delay between phytoplankton and bacterial responses, while the magnitude and sign of the correlation coefficient at that lag reflect the strength and direction of the association. Positive lags suggest that changes in chlorophyll *a* concentration precede changes in bacterial cell counts, whereas negative lags imply the opposite.

## 3 Results

### 3.1 Sampling time and alkalinity gradient drive beta diversity structure

**Figure 1:**
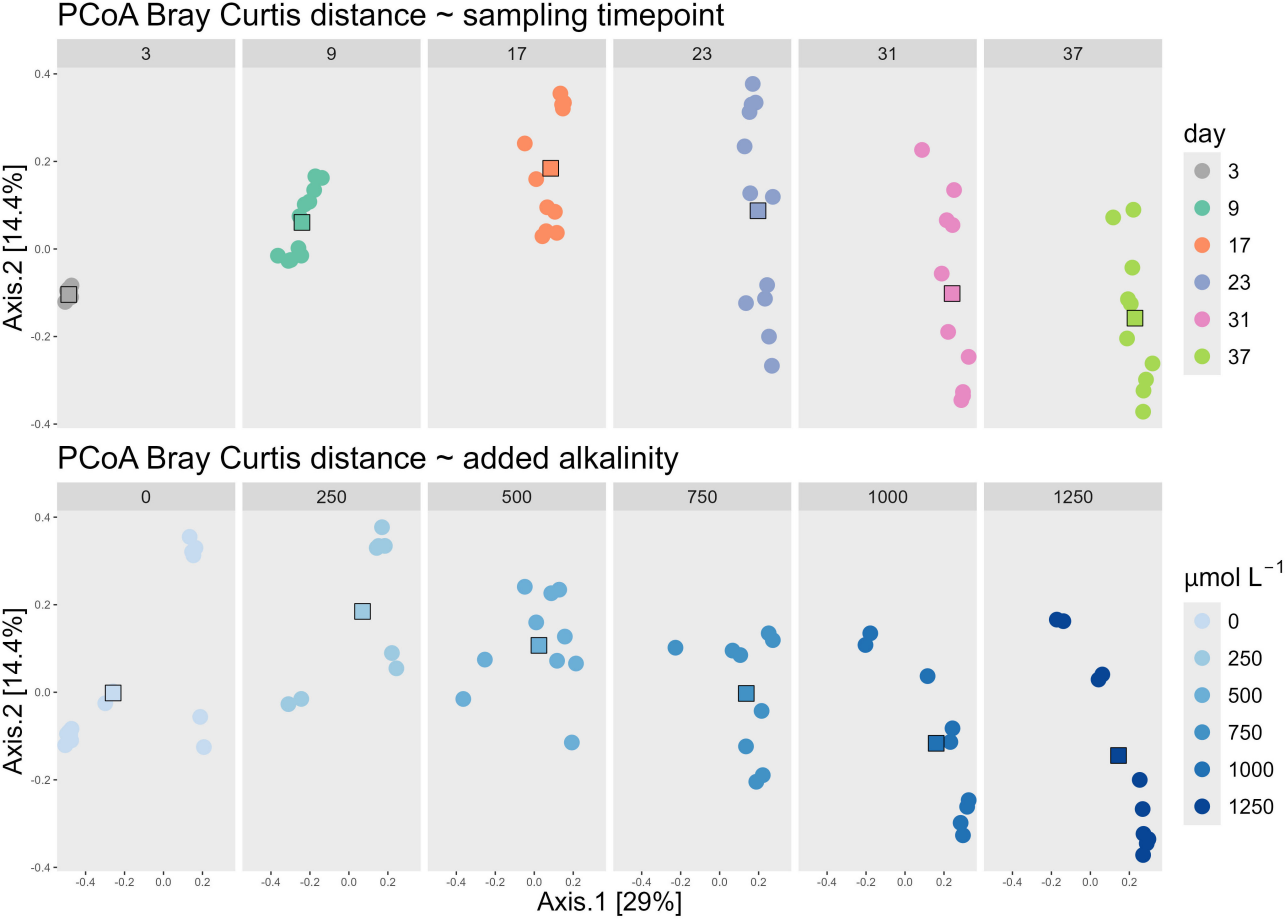
PCoA of all samples using the Bray Curtis dissimilarity matrix, faceted by the day of the different groups of the sampling time point (upper panels) and faceted by the different groups of the added alkalinity concentration (lower panels). Both the upper and lower panels are the same PCoA, which can be seen cumulated on the left panel of **Figure 2**. The faceting into each sampling timepoint group and alkalinity group, is done to visualize how the centroids move along the dominant two axes. The centroids of the increasing sampling timepoints move towards the right along the x-axis, while the centroids of increasing alkalinity addition down on the y-axis.

Alkalinity addition influenced beta diversity, but the sampling time point was the primary driver of variation in bacterial community composition. The PCoA based on Bray–Curtis dissimilarities illustrated the factors driving the changes in bacterial community composition across all 65 samples taken during the experiment. Axis 1 explained 29.0% of the variance, while Axis 2 accounted for 14.4%, resulting in a combined explained variance of 43.4%. Visualizing the data by facets of sampling time and alkalinity treatment revealed trends along the gradients of both parameters (**Figure 1**): Axis 1 was associated with sampling time, as sample centroids shifted progressively along the positive direction of this axis over the course of the experiment—from day 3 (centroid: −0.49) to day 37 (centroid: 0.22). In contrast, axis 2 was closely aligned with the gradient of added alkalinity. Centroids of samples with increasing alkalinity additions shifted downward along the y-axis, from 0.18 (250 µmol/L) to −0.14 (1250 µmol/L). The centroid of the untreated control (0 µmol/L) deviated from this trend, positioned near 0.00 on the y-axis, suggesting a weaker association with this axis.

**Figure 2:**
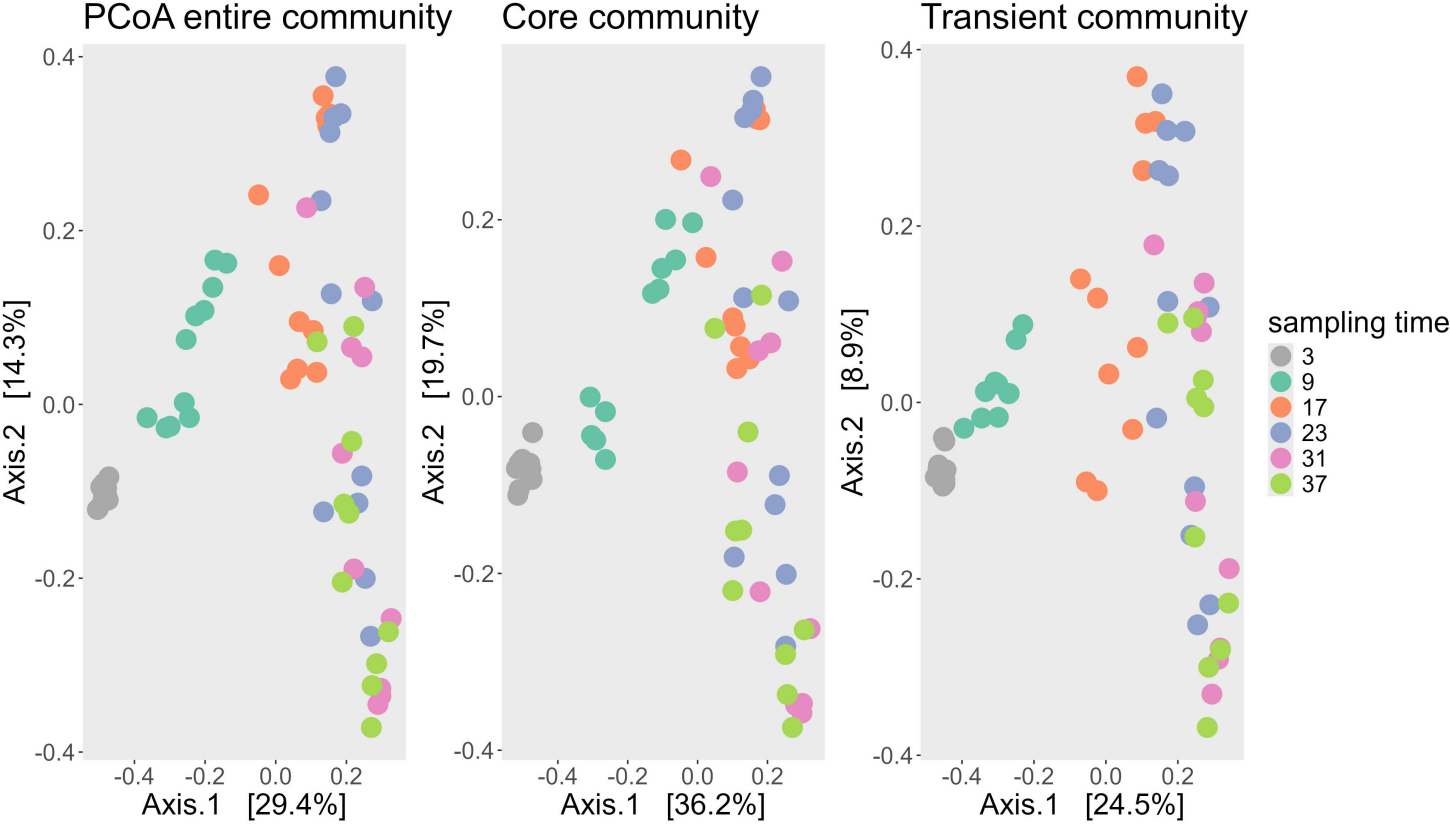
PCoA ordination of bacterial communities based on Bray-Curtis dissimilarities for the entire community (left), the core microbiome (center), and the transient microbiome (right). The core community includes ASVs present in at least 50% of samples (139 ASVs), while the transient community comprises all other ASVs (2,169 ASVs). Each point represents one sample, color-coded by sampling day. The first axis explains the majority of variance in all three ordinations and corresponds primarily to sampling time.

A similar trend was observed in the ordination of the core and transient fractions of the bacterial community (**Figure 2**). The ordination patterns across the PCoA plots of all ASVs, the core ASVs (present in ≥50% of samples), and transient ASVs (present in <50% of samples) were largely consistent, with sampling time again aligning with axis 1 and alkalinity with axis 2. However, the proportion of explained variance differed. In the core community, the two main axes accounted for 55.9% of total variability, whereas in the transient community they explained only 33.4%, indicating that the core microbiome was more influenced by the experimental conditions. In contrast, the transient community exhibited greater stochasticity.

### 3.2 Patterns in the taxonomic community composition

**Figure 3:**
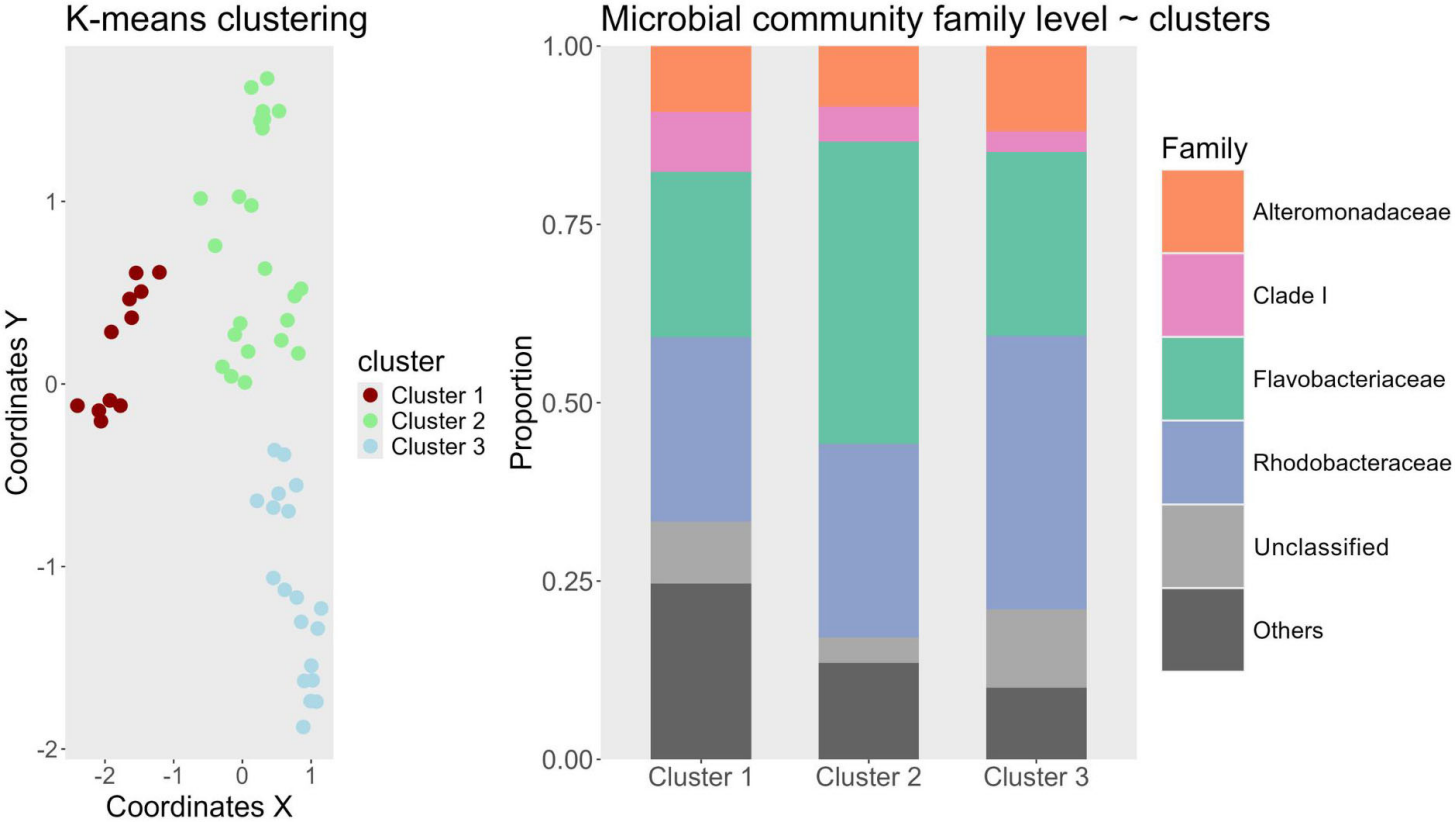
Left: K-means Clustering Results. The coordinates on the Y and X axes originate from a PCoA ordination (Supplement). Different clusters identified by the algorithm are color-coded and detailed in the legend. Samples from the initial sampling were excluded from the analysis as they represent baseline samples from each mesocosm without any treatment. Right: Stacked bar plot of the most common bacterial families found in the clusters. X-axis shows one of the three different clusters and the Y-axis shows the proportion between 0 and 1. Colors are indicative of bacterial taxonomic group at family level depicted in the legend to the right. “Others” are all taxa which fall under a threshold of 15% of the overall community.

With k-means clustering, the 65 community samples were classified into three distinct clusters (**Figure 3**). The clustering results revealed that sampling time was the dominant driver of taxonomic variation. Samples collected during the first week of the experiment primarily fell into Cluster 1, while those from later time points grouped into clusters 2 and 3 (**Figure 3**). Notably, most samples from the final week were assigned to cluster 3, with only 2 samples assigned to cluster 2. The chi-square test of independence confirmed that sampling time had the strongest association with cluster assignment (p = 0.00007), followed by alkalinity treatment (p = 0.002), whereas dilution treatment had no significant association (p = 0.756). While alkalinity was statistically significant, its influence was secondary to sampling time point. The SIMPER analysis identified the ASVs that contributed most of the dissimilarity between clusters (678 ASVs). Across all clusters, dominant families included Rhodobacteraceae, Flavobacteriaceae, and Alteromonadaceae, though their relative abundances varied (**Figure 3**). Cluster 1 showed a relatively even distribution of taxonomic families, whereas cluster 3 was dominated by Rhodobacteraceae (38.3%), indicating a temporal trend toward reduced evenness and increased dominance. The “Others” category, comprising families contributing <15% to the total community, declined from cluster 1 (24.6%) to cluster 3 (10.1%), further suggesting a narrowing of taxonomic diversity. The SAR11-associated Clade I decreased from cluster 1 (8.4%) to cluster 3 (2.9%), while Flavobacteriaceae peaked in relative abundance in cluster 2 (42.4%). To complement the taxonomic analysis, alpha diversity was assessed using richness, Shannon, and Simpson indices (see supplement for plot). Alpha diversity declined from cluster 1 to cluster three across all matrices. Cluster 1 consistently exhibited the highest diversity (53.81 Richness, 3,51 Shannon, 0.95 Simpson), followed by cluster 2 (53.12 Richness, 3,30 Shannon, 0.93 Simpson). In contrast, cluster 3 showed a marked decline in both richness and evenness (48.36 Richness, 3,24 Shannon, 0.93 Simpson).

### 3.3 Effects of alkalinity, sampling time and dilution on microbial community

The PERMANOVA results indicated that both alkalinity addition and sampling time had significant effects on bacterial community composition, while dilution treatment had no statistically detectable effect (**Table 1**). The sampling time had the strongest effect on the community composition. *Post hoc* pairwise comparisons revealed that most sampling days differed significantly from one another (**Table 2**). Notably, samples from day 9 differed significantly from those of all other time points (p < 0.05), and samples from day 17 differed significantly from samples taken on all other days except from day 23. Toward the end of the experiment, the number of significant differences between time points decreased, particularly between samples taken on days 31 and 37, no significant differences were detected. Considering the alkalinity treatments, only the highest addition level (Δ1250 µmol/L) differed significantly from the control (Δ0 µmol/L) after the Bonferroni correction (p = 0.045; **Table 3**).

**Table 1:** Centroid based PERMANOVA with Bray Curtis dissimilarity.

Parameter	p-value	Significance	SS	R²	df
Alkalinity	0.001	*	1.82	0.14	5
Incubation Time	0.012	*	3.72	0.29	4
Treatment	0.323				

Significance is indicated by a star if p ≤ 0.05.

**Table 2:** *Post Hoc* test sampling time on a Bray Curtis dissimilarity matrix.

Pairs	P-value	P-adjusted	Significance
9 *vs* 17	0.001	0.01	*
9 *vs* 23	0.001	0.01	*
9 *vs* 31	0.001	0.01	*
9 *vs* 37	0.001	0.01	*
17 *vs* 23	0.069	0.69	
17 *vs* 31	0.001	0.01	*
17 *vs* 37	0.001	0.01	*
23 *vs* 31	0.033	0.33	
23 *vs* 37	0.004	0.04	*
31 *vs* 37	0.506	1	

P-value adjusted indicates a p-value after the Bonferroni correction method. Significance is indicated by a star if p ≤ 0.05.

**Table 3:** *Post hoc* test added alkalinity.

Pairs	P-value	P-adjusted	Significance
0 *vs* 250	0.192	1	
0 *vs* 500	0.381	1	
0 *vs* 750	0.006	0.09	
0 *vs* 1000	0.044	0.66	
0 *vs* 1250	0.003	0.045	*
250 *vs* 500	0.251	1	
250 *vs* 750	0.156	1	
250 *vs* 1000	0.516	1	
250 *vs* 1250	0.481	1	
500 *vs* 750	0.172	1	
500 *vs* 1000	0.29	1	

P-value adjusted indicates a p-value after the Bonferroni correction method. Significance is indicated by a star if p ≤ 0.05.

### 3.4 Alkalinity induced effects on predicted functional pathways

**Figure 4:**
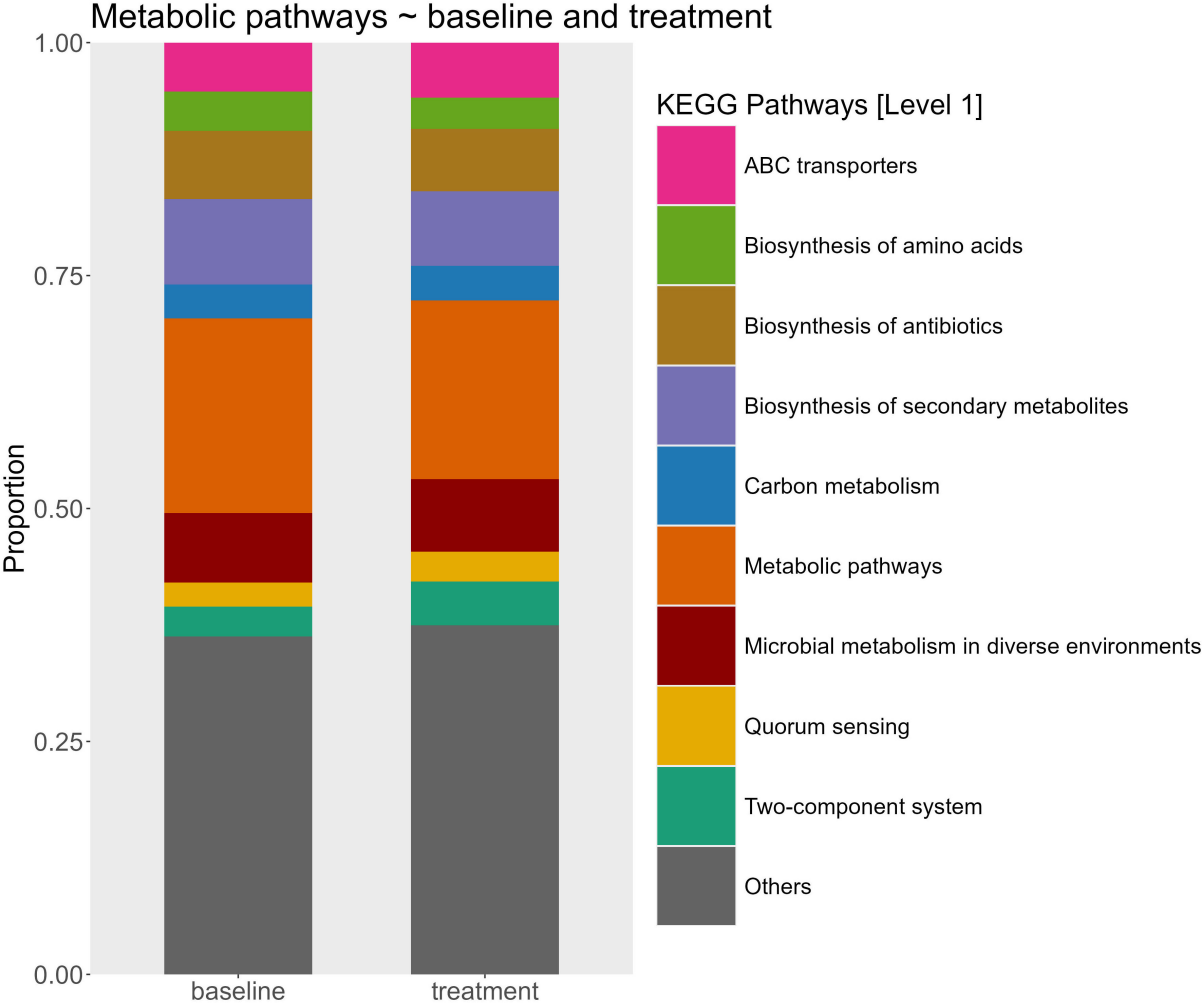
Relative abundance of predicted microbial metabolic pathways at the KEGG category level 1 between baseline mesocosms (n=12) and treated mesocosms (n = 53). The stacked bar plots represent the proportional contribution of major pathway categories inferred from community functional profiles.

Differences in the predicted functional pathways between groups of alkalinity addition and sampling time point were tested using PERMANOVA. This analysis revealed significant effects of both sampling time (p = 0.001, R² = 0.29) and added alkalinity (p = 0.016, R² = 0.14) on the predicted functional pathways of the microbial communities. *Post hoc* pairwise comparison of sampling time point groups showed that only the predicted functional pathways from the first time point (day 3) differed significantly from groups of later sample time points in the experiment (**Table 4**). In contrast, pairwise comparisons between groups of the different alkalinity levels did not yield significant differences after correction for multiple testing (**Table 5**). A further SIMPER analysis on the functional pathways between baseline samples and treated mesocosm samples was used to visualize differences (**Figure 4**). Relative abundances of KEGG pathways exhibited only modest variation between baseline samples and treatment conditions. The most abundant category, “Metabolic pathways,” showed a slight decrease from 20.89% at baseline to 19.18% in treatment groups. Minor changes were also observed for pathways such as “Biosynthesis of antibiotics” (increase from 7.99% to 9.17%) and “Biosynthesis of amino acids” (decrease from 4.23% to 3.36%), other pathways like “Quorum sensing (2.61% to 3.22%)” and “ABC transporters (5.26% to 5.90%)” remained largely unchanged. Carbon metabolism pathways similarly displayed no changes in the relative abundance between baseline and treated samples with a small increase from 3.63% to 3.71%. Likewise, the dominant “Others” category (36.27%–37.48%) also remained stable.

**Table 4:** Centroid based PERMANOVA with Bray Curtis dissimilarity to test differences in metabolic pathways and predicted functionality between baseline and treatment mesocosms.

Parameter	P-value	Significance	SS	R²	df
Added Alkalinity	0.015	*	0.011	0.05	1
Sampling Time	0.001	*	0.088	0.417	5

The symbol * means that the p-value in this row is significant and thus smaller than 0.05.

**Table 5:** *Post hoc* test of sampling time point and alkalinity addition on pathways and predicted functionality.

Sampling timepoint				Added alkalinity			
Pairs	P-value	P-adjusted	Significance	Pairs	P-value	P-adjusted	Significance
3 *vs* 9	0.001	0.015	*	0 *vs* 500	0.04	0.6	
3 *vs* 17	0.001	0.015	*	0 *vs* 250	0.027	0.405	
3 *vs* 23	0.001	0.015	*	0 *vs* 1250	0.029	0.435	
3 *vs* 31	0.001	0.015	*	0 *vs* 1000	0.279	1	
3 *vs* 37	0.001	0.015	*	0 *vs* 750	0.085	1	
9 *vs* 17	0.478	1		500 *vs* 250	0.46	1	
9 *vs* 23	0.057	0.855		500 *vs* 1250	0.259	1	
9 *vs* 31	0.032	0.48		500 *vs* 1000	0.481	1	
9 *vs* 37	0.008	0.12		500 *vs* 750	0.727	1	
17 *vs* 23	0.164	1		250 *vs* 1250	0.162	1	

The symbol * means that the p-value in this row is significant and thus smaller than 0.05.

### 3.5 Effects of alkalinity on total bacterial cell counts and chlorophyll interactions

To assess whether alkalinity addition affected bacterial abundance, we calculated the cumulative bacterial cell counts for each mesocosm. The results revealed a non-linear response: the highest cumulative bacterial cell were observed at the 500 µmol/L treatment (31,425 cells/µL), whereas counts declined at higher alkalinity additions. Specifically, at 1000 and 1250 µmol/L alkalinity addition, total counts fell to 13,459 and 11,765 cells/µL, respectively. Both high alkalinity treatments had cumulative bacterial cell counts markedly lower than the control mesocosms (16,413 cells/µL). Bacterial cell count peaks were observed in seven mesocosms with alkalinity treatments ranging from 0 to 750 µmol/L, typically reaching between 2000 and 4000 cells/µL. In the control and 250 µmol/L mesocosms, peak abundance occurred on day 20, whereas in the 500 µmol/L treatments, peaks were delayed until day 28 (**Table 6**). Among the two mesocosms with 750 µmol/L alkalinity addition only the delayed dilution (DEL) treatment exhibited a clear peak, while the immediate dilution (IMM) treatment did not. The DEL mesocosm at 1000 µmol/L exhibited a steady increase in bacterial cell counts, suggesting the onset of a peak. The other high-alkalinity mesocosms (1000 IMM and both 1250 µmol/L alkalinity addition) did not exhibit comparable peaks at all.

**Table 6:** Bacterial cell count and chlorophyll peaks.

Alkalinity addition	Cell count peak average	Chlorophyll peak average
0	20	11
250	20	11
500	28	9
750	23	12
1000	25	18
1250	13	22

The Alkalinity addition column marks the Alkalinity addition group, while the average of the two diluent treatments is calculated for cell counts and chlorophyll.

Chlorophyll measurements mirrored this pattern, with peak concentrations likewise being delayed at higher alkalinity levels. The six mesocosms with alkalinity additions from 0 to 500 µmol/L had chlorophyll peaks on day 10, whereas in mesocosms with higher alkalinity addition, peak concentrations occurred at day 17 on average. Among the seven mesocosms that exhibited distinct bacterial peaks, the bacterial response lagged behind the chlorophyll peak by an average of 13.5 days (**Table 6**). In the highest alkalinity treatments (1000 and 1250 µmol/L), the observed bacterial peak preceded the chlorophyll peak. Visual inspection of the chlorophyll trends showed no detectable peak in the mesocosms with 1000 and 1250 µmol/L alkalinity addition.

**Figure 5:**
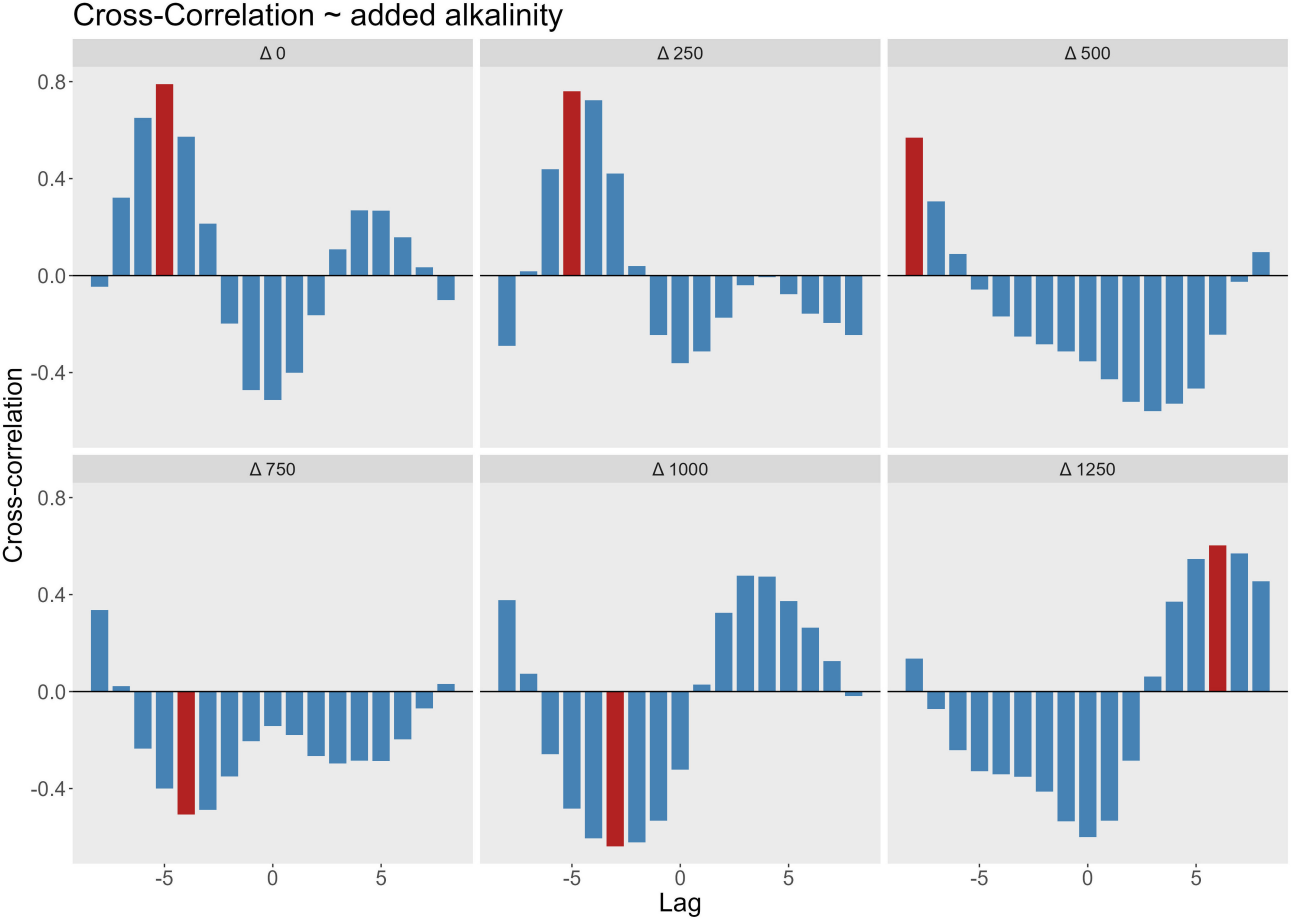
Cross-correlation between bacterial cell counts and chlorophyll concentrations at varying levels of added alkalinity (Δ0–Δ1250 µmol/L). Each panel represents a different alkalinity treatment level, showing the cross-correlation coefficient (y-axis) at different time lags 1 lag = 2 days, as sampling was done every second day. Positive lags indicate chlorophyll leads bacteria; negative lags indicate bacterial dynamics precede chlorophyll. Red bars highlight the lag with the highest absolute correlation value in each panel.

**Figure 6:**
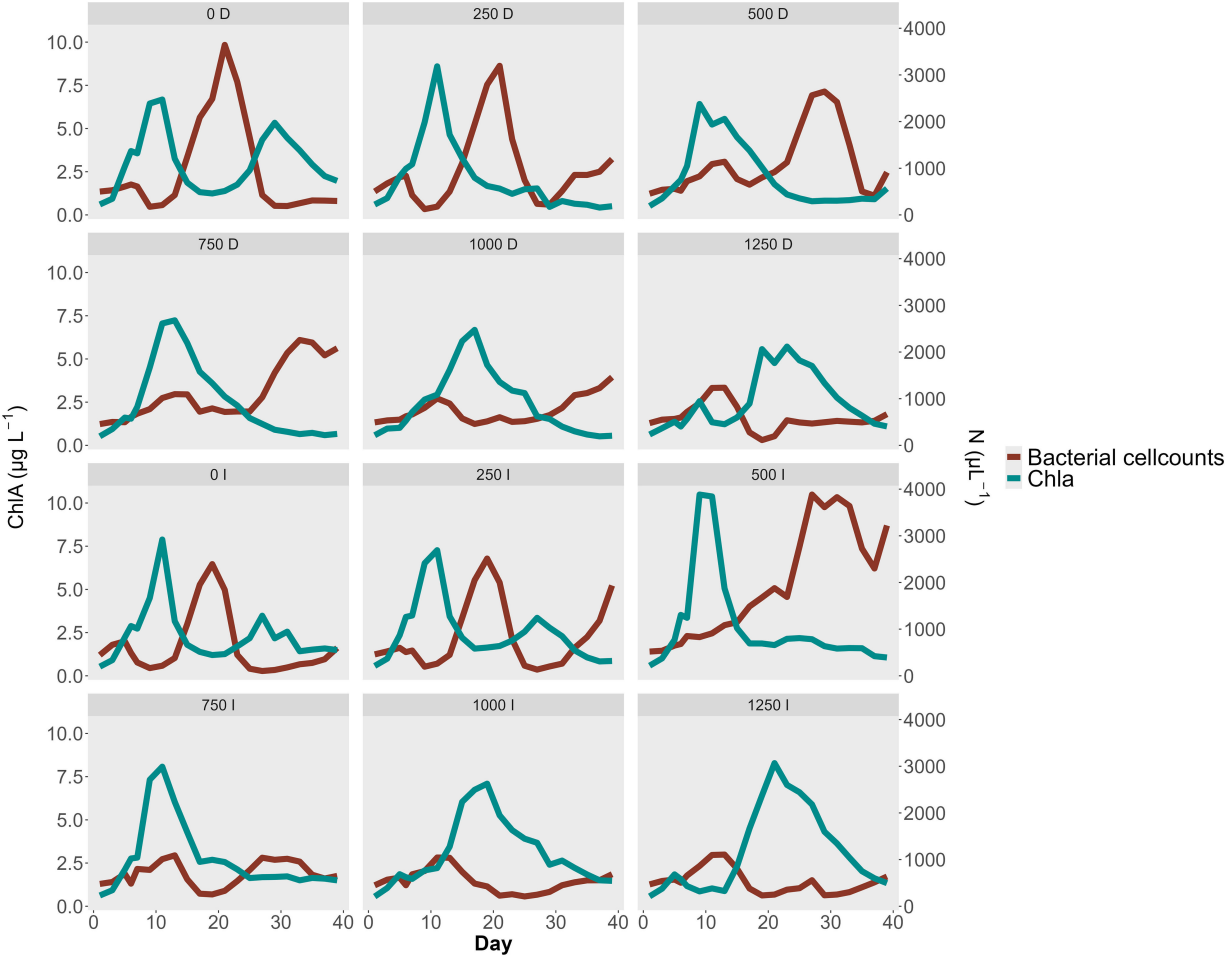
Bacterial Cell count N µL-1 and Chlorophyll concentration µg L-1 during the experiment. Each panel shows one Mesocosm with a short text describing the Treatment. The number shows the level of alkalinity addition, with “D” = Diluted treatment and “I” = Immediate treatment. The plot shows the inverse corelated growth dynamics between bacteria and chlorophyll. Higher alkalinity concentrations shift chlorophyll blooms to later points in the experiment which is reflected in an even later bacterial cell abundance peak. The treatment started on day 4.

To further investigate the relationship between phytoplankton and bacterial abundance, we performed a cross-correlation analysis (**Figure 5**). In low to moderate alkalinity treatments (0–250 µmol/L), the strongest positive correlation occurred at lag −5 (10 days), with a maximum correlation coefficient of 0.8, showing that bacterial peaks lagged behind phytoplankton peaks by 10 days. In the mesocosms with 500 µmol/L alkalinity addition, the maximum correlation was observed at lag −8 (16 days), with a decline in the correlation coefficient to 0.57. For the 750 µmol/L alkalinity addition treatments, the highest correlation was negative and occurred at lag −4, corresponding to a decline in chlorophyll *a* concentrations from days 10 to day 20, followed by increased bacterial abundance from days 20 to day 25 (**Figure 6**). A Similar inverse correlation was observed in the mesocosms with 1000 µmol/L alkalinity addition. In the 1250 µmol/L mesocosms, the strongest correlation was positive at lag +5. This was associated with an early increase in bacterial counts (days 5–13), followed by a rise in chlorophyll (days 13–20), and then a concurrent decline in both. In all mesocosms, the correlation at lag 0 was negative, indicating an inverse relationship between bacterial cell abundance and chlorophyll *a* concentration. Bacterial counts increased immediately following the filling in all mesocosms. In mesocosms with no or low added alkalinity (0–250 µmol/L), this increase was short-lived and diminished soon after alkalinity was added. In contrast, at higher alkalinity levels, bacterial growth persisted for longer, while phytoplankton growth was delayed.

## 4 Discussion

This study is part of a broader mesocosm experiment conducted by the RETAKE consortium of CDRmare, aimed at investigating the ecological effects of OAE, with a specific focus on bacterial communities. To date, relatively few studies have examined ecotoxicological effects of OAE on bacterial communities. As a result, there are considerable knowledge gaps present that need to be investigated. In this study we address these knowledge gap by investigating how OAE influences bacterial communities qualitatively and quantitatively.

### 4.1 Effects of OAE on bacterial taxonomy and predicted functionality

The results of the taxonomic analysis indicate that the bacterial communities in the mesocosms were only marginally affected by the alkalinity treatments, with the most observable shifts between communities being explained by the sample time point. We hypothesized that the beta diversity of the bacterial communities would align to the imposed alkalinity gradient. This experiment tested the effects of unequilibrated alkalinity addition. Unequilibrated alkalization leads to pH increases. It is well-established that pH is a major environmental determinant shaping microbial community composition in marine systems (Krause et al., 2012). Our analysis was limited to beta diversity metrics and did not allow for the identification of alkaliphilic taxa. We focused on beta diversity measures as it was not feasible to detect single alkaliphilic bacterial taxa using 16S rRNA gene amplicon sequencing, as it only provides taxonomic resolution up to the genus level (Zhang et al., 2023). The pH tolerance of bacterial taxa can vary considerably at the species and strain level (Kim and Ndegwa, 2018). The most abundant families identified in the analysis illustrate this point. Rhodobacteraceae and Flavobacteriaceae are metabolically versatile and known to thrive across broad pH ranges, in some cases spanning from pH 6 to 9 (Wong et al., 2017; Ma et al., 2022).

It was unexpected to see that the temporal dynamics played such a dominant role in shaping the bacterial community composition. When the three identified clusters are viewed in chronological order and compared with established site-specific dynamics, the observed taxonomic shifts align with the known spring succession patterns at Helgoland Roads. Teeling et al. (2016), who characterized bacterioplankton dynamics in this region over multiple spring seasons, reported trends such as an increase in Rhodobacteraceae and a decline in the SAR11 clade—patterns that are also evident in our data. Other patterns shared by both datasets include an increasing relative abundance of Alteromonadales and Bacteroidetes, and a decreasing abundance of Cellvibrionales (see supplement). This finding suggests that the marine bacterial community was resilience to alkalization in the experiment and that the seasonal succession was the main driver for the observed community shifts.

The resilience of bacterial communities to alkalization is further supported by the analysis of the prediction of functional metabolic pathways. Predicted functional profiles showed no significant increase or restructuring of carbon metabolic pathways in treated mesocosms compared to baseline controls. We hypothesized that mesocosms receiving alkalinity additions would exhibit a detectable increase in the relative abundance related to carbon metabolism, due to the anticipated reduction dissolved carbon availability. However, the results did not support this hypothesis. This highlights the high degree of functional stability within the microbial communities during the experiment, even when the communities themselves were different in structure. This is likely due to functional redundancy within marine microbial communities. Different communities can possess overlapping capacities for carbon metabolism. Thus, even if succession leads to shifts in the bacterial composition, the presence of redundant genes can lead to similar predicted functional outputs (Puente-Sánchez et al., 2024).

Our finding that bacteria are resilient to alkalinity addition contrasts with the results of Ren et al. (2021), which, to our knowledge, is the only other study to investigate impacts of OAE on bacterial communities. Ren et al. (2021) used olivine to increase alkalinity and the levels reached were ~200 µmol/L. Despite far lower alkalinity levels than compared to the OAE study presented here, they observed statistically significant alterations in the community structure in particle-associated bacterial communities. Our study did not separate particle-attached and free-living bacterial communities and instead analyzed the bacterial community as a whole. One could argue that an effect on the entire community would be expected under higher alkalinity conditions, given that the particle-attached community is altered by far lower alkalinity levels. However, our data did not support this assumption. Only a small fraction of bacteria in the water column are particle-attached (Cho and Azam, 1988; Mestre et al., 2017). As our analysis did not separate the bacteria into particle-attached and free-living communities, the relative abundance of the free-living bacteria possibly obscures the detection of changes in the particle-attached bacterial community. The difference may also be caused by the different substances used for alkalization. The study by Ren et al. (2021) increased the alkalinity with olivine, which is a particle-based OAE approach. It is possible that the observed changes in the particle-attached bacterial community originated from the olivine as a particle and not from the increased alkalinity.

OAE is a relatively new research topic, and studies on its ecological impacts, particularly on bacterial communities, are scarce. In contrast, the effects of ocean acidification have been extensively studied. Given that alkalization and acidification represent opposite shifts along the same pH continuum, it is reasonable to assume that their biological impacts may exhibit parallels for microbial communities. We argue that, for now, studies documenting the effects of ocean acidification on bacterial communities are the best available comparison to establish some contrast for our results. For example, a mesocosm study conducted in the Gullmar Fjord, Sweden, employed two acidification treatments with five replicates and found that changes in the bacterial community were largely masked by seasonal succession (Langer et al., 2017). This aligns closely with our findings, where sampling time emerged as the dominant driver in the community structure. Similarly, another acidification study using a gradient-based experimental design that is comparable to ours, also concluded that temporal dynamics were the main factor shaping microbial communities (Sperling et al., 2013). All these studies used large-scale mesocosms and found that bacterial community shifts were primarily driven by time, not by the imposed pH changes, suggesting a general resilience of bacterial communities to both acidification and alkalization.

This apparent resilience is, however, not universal. There is a highly relevant study, also conducted with water from Helgoland Roads and it revealed statistically significant effects of moderate acidification on bacterial communities (Krause et al., 2012). There is a significant methodological distinction that likely explains these different conclusions. The study by Krause et al. (2012) included seasonal replication and thus was able to disentangle pH effects from seasonal succession, unlike the studies by Langer et al. (2017) and Sperling et al. (2013), which were conducted within a single season. Conducting the same experiment across multiple seasons allows researchers to control for seasonal succession and thereby better isolate the effects of acidification. A similar approach may be necessary for studying the effects of alkalization, suggesting that experimental designs should aim to isolate the impact of alkalinity enhancement in order to detect potential effects on bacterial communities.

### 4.2 Effects of OAE on bacterial cell abundance

Although our amplicon sequencing results indicated that bacterial community composition was largely resilient to alkalinity treatments, our quantitative analysis revealed differences in the cell abundance. Bacterial cell counts exhibited pronounced shifts in response to OAE, particularly in the timing and magnitude of abundance peaks. Mesocosms that were receiving the highest level of alkalinity additions (1250 µmol/L) exhibited smaller cumulative bacterial cell counts. This supports our hypothesis that high levels of unequilibrated alkalinity might suppress bacterial abundance due to altered carbon fluxes. However, these findings must be interpreted with caution. In mesocosms with high alkalinity treatments, bacterial cell counts peaks were delayed. The experiment may have ended before bacterial abundance reached its peak, potentially underestimating cumulative cell counts in mesocosms with higher alkalinity levels, compared to what would have occurred with a longer experiment. A consistent trend across treatments was observed: bacterial cell count peaks generally followed those of chlorophyll *a* concentrations, with lag times increasing at higher alkalinity levels. This lagged relationship suggests an indirect effect of OAE on bacteria, mediated through its influence on phytoplankton. Alkalinity additions appears to delay phytoplankton growth, which in turn postponed bacterial abundance peaks due to the reduced availability of organic carbon. This dynamic is consistent with established bloom patterns, where heterotrophic bacteria respond and recycle organic matter produced by phytoplankton (Cunliffe et al., 2009; Teeling et al., 2012).

This pattern of delayed blooms mirrors findings from ocean acidification experiments. In a mesocosm experiment by Newbold et al. (2012) the peaks in bacterial abundance occurred two days earlier under acidified conditions, while phytoplankton peaks were advanced by four days relative to controls. In our case, alkalinity additions delayed both chlorophyll *a* and bacterial peaks, suggesting a mirrored response across the pH spectrum. The study by Newbold et al. (2012), together with our findings, highlight that quantitative shifts in bacterial abundance occur in response to changes in phytoplankton bloom timing—being advanced under acidified conditions and delayed under alkalization treatments. However, this pattern may only be accurate for seasons with phytoplankton blooms. Notably, Newbold et al. (2012) intentionally induced a phytoplankton bloom to investigate the effects of acidification, whereas our study was conducted in March to coincide with the natural spring bloom period.

Increased alkalinity levels did not appear to inhibit bacterial growth. At the beginning of the experiment, bacterial cell counts increased in all mesocosms and only began to decline with the onset of the phytoplankton bloom. This suggests that bacterial abundance is less affected by alkalinity increases than by phytoplankton blooms. These temporal shifts may be explained by differences in adaptability between bacteria and phytoplankton. Bacteria generally have shorter generation times, usually measured on the scale of minutes to hours, compared to the days required for phytoplankton replication (Furnas, 1990; Laws, 2013). This allows bacteria to respond more rapidly to environmental changes (Kirchman, 2016). However, this competitive advantage is limited, as bacteria are ultimately dependent on phytoplankton-derived organic matter. As phytoplankton adapts and blooms emerge, bacterial cell counts decline, and their abundance peaks occur later, after the phytoplankton bloom. At lower alkalinity levels (~500 µmol/L), phytoplankton is less affected, and peaks in chlorophyll *a* and bacterial abundance occur without temporal delay compared to untreated controls. We conclude that the observed effects of alkalinity on bacterial abundance are not direct, but instead arise indirectly through interactions with phytoplankton.

## 5 Conclusion

Bacterial communities demonstrated resilience to alkalinity enhancement within the framework of the mesocosm experiment. Temporal succession emerged as the primary driver of shifts in bacterial community composition. Bacterial abundance was indirectly affected by interactions with phytoplankton, with alkalinity additions of 500 µmol/L representing a potential threshold with no observable effect. This study emphasizes the value of combining amplicon sequencing with some form of quantitative analysis to gain a more comprehensive understanding of microbial dynamics (Thomas et al., 2024). We recommend implementing such combined approaches in future studies wherever feasible. Effects from OAE on the bacterial community are obscured by seasonal changes. We suggest that future experiments isolate effects from alkalinity similarly to Krause et al. (2012) by conducting experiments during multiple seasons in temperature-controlled settings. It remains unclear whether previously observed changes in particle-associated bacterial communities were caused by increased alkalinity or by the addition of particulate matter. To clarify this, future experiments should separately analyze free-living and particle-attached communities, while accounting for the type of substance used for alkalization. If particulate substances are used to increase alkalinity, the setup must include controls for particle addition with non-alkalizing particles. The observed quantitative effects from alkalinity on the bacterial community was connected to the phytoplankton dynamics and their interactions with bacteria. To further isolate effects of alkalinity on the bacterial community, filtration of the seawater could be considered to remove primary producing algae. For the future of OAE application it needs to be highlighted that there are many uncertainties. This study details how to identify changes in the bacterial community during OAE. Further studies and field trials are needed to investigate interactions with other organisms and to assess the effects of different alkalinizing agents, in order to evaluate the feasibility of large-scale OAE implementation.

## Data Availability

The datasets presented in this study can be found in online repositories. The names of the repository/repositories and accession number(s) can be found below: https://www.ncbi.nlm.nih.gov/, PRJNA1245293.
