# Supplementary material for: The effect of ocean alkalinity enhancement on pelagic bacterial communities: focus points derived from a mesocosm experiment
Source: Front Microbiomes. 2025 Aug 11;4:1606890. doi: 10.3389/frmbi.2025.1606890 (PMC12993613; doi:10.3389/frmbi.2025.1606890)
Supplement: Supplementary file 1 [file Supplementaryfile1.docx]

Supplementary Material

# Experimental design


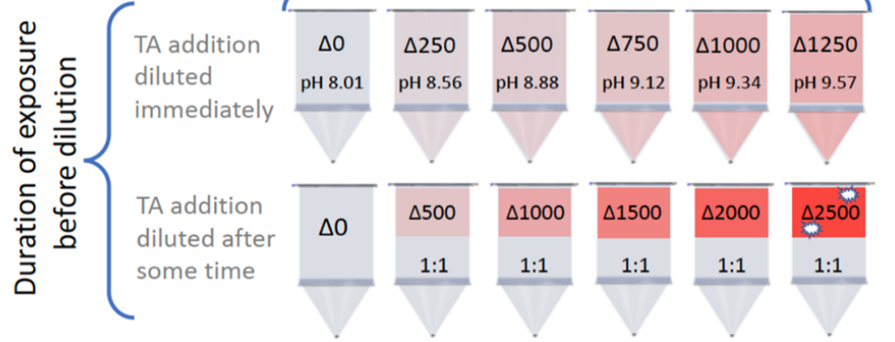


**Supplementary Figure 1.** Treatment table. This schedule shows the 12 different treatments of the RETAKE Helgoland mesocosm campaign. In the upper row the Immediately diluted treatment is illustrated in the six used mesocosms, while the lower column depicts the delayed dilution with double the added alkalinity concentration at the top layer of the mesocosm for three days. The upper number shows the amount of alkalinity added in µmol L-1 and the lower number shows the expected pH value based on modeling of the water chemistry with CO2sys (Humphreys et al. 2022). The spots in the highest dilution treatment symbolize an expected secondary precipitation. As the secondary precipitation happened to sparsely during the experiments its effects on the microbiome could not be analysed in the frame of this analysis.

# Additional plots for the microbial analysis


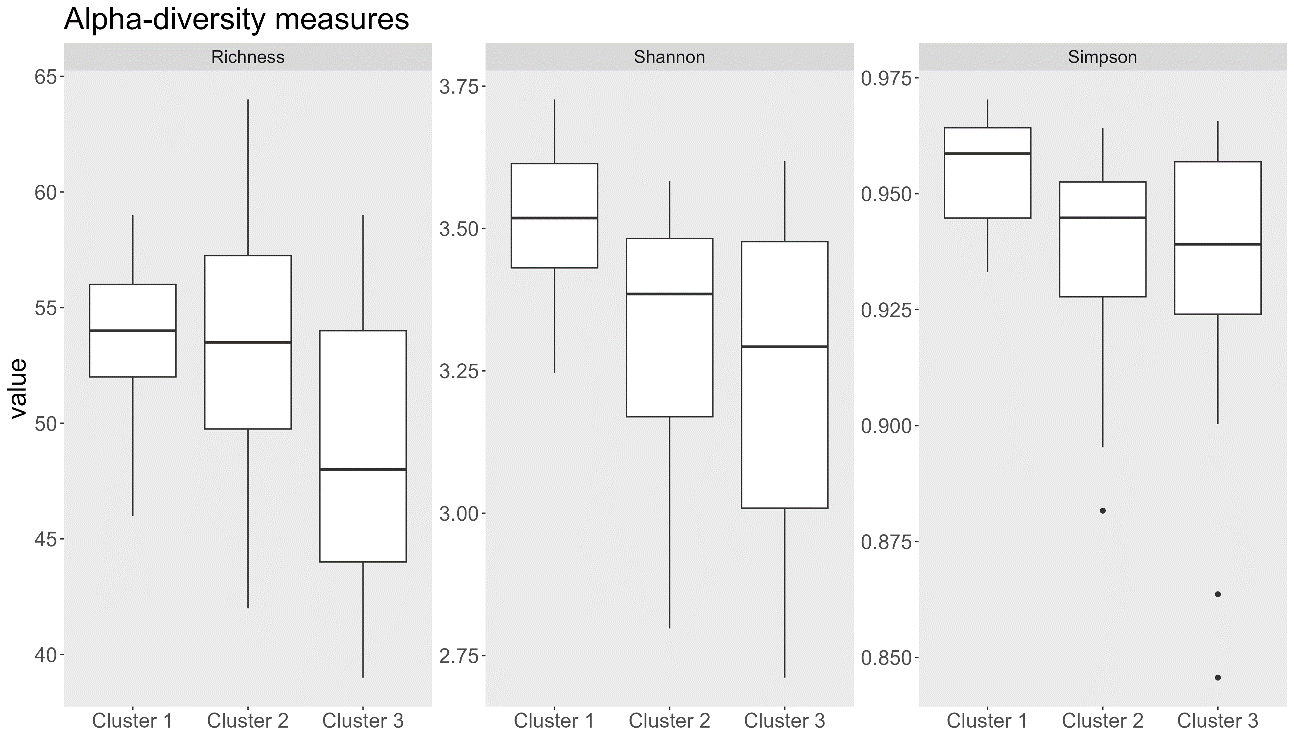


**Supplementary Figure 2.** Alpha diversity indices of bacterial communities included in the SIMPER analysis. The communities shown here represent the subset of ASVs that cumulatively explain 70% of the dissimilarity between clusters identified through k-means clustering. The x-axis indicates the three clusters, while the y-axis shows the values of three diversity indices: Richness, Shannon, and Simpson.

**
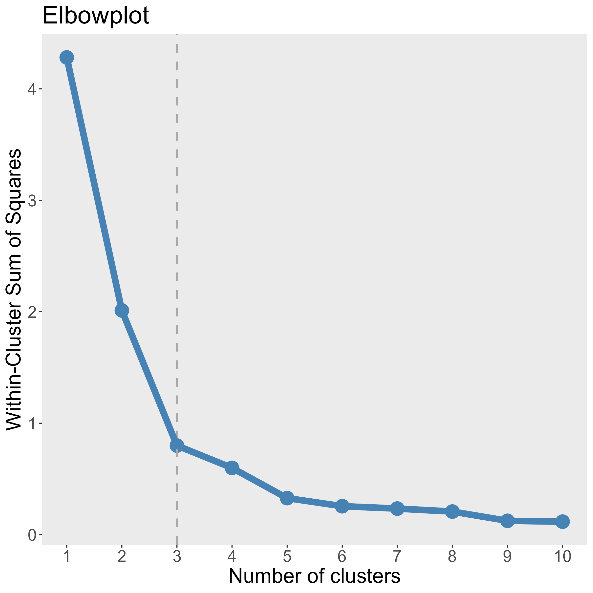

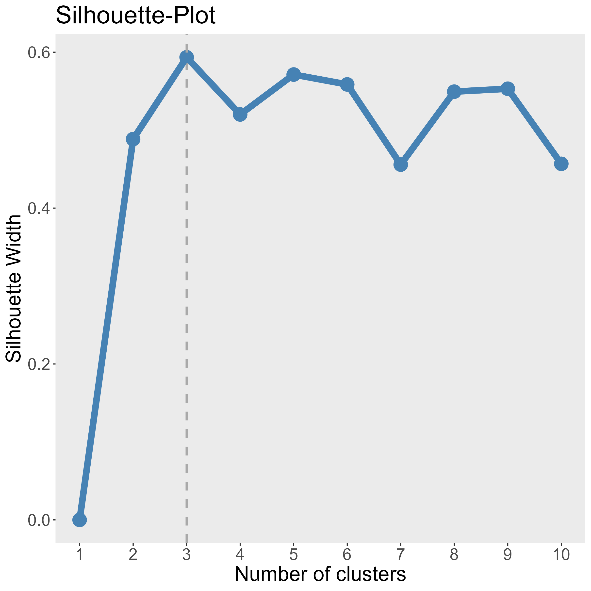
**

**Supplementary Figure 3.** Elbow and Silhouette Plots. The Elbow plot (left), and the Silhouette plot (right)serve as visual aids in determining the optimal number of clusters for the K-means algorithm. The Elbow method helps identify the point of diminishing returns in variance explained, while the Silhouette method assesses the quality of clustering. Both plots converge on the ideal number of clusters, indicated by the dashed line, which in this case is 3.

**Supplementary Figure 4.** Stacked bar plots proteobacteria: X axis shows one of the three different clusters and the Y axis shows the proportion between 0 and 1. Colors are indicative of proteobacterial Orders depicted in the legend to the right. “Others” are all Orders which fall under a threshold of 10%.


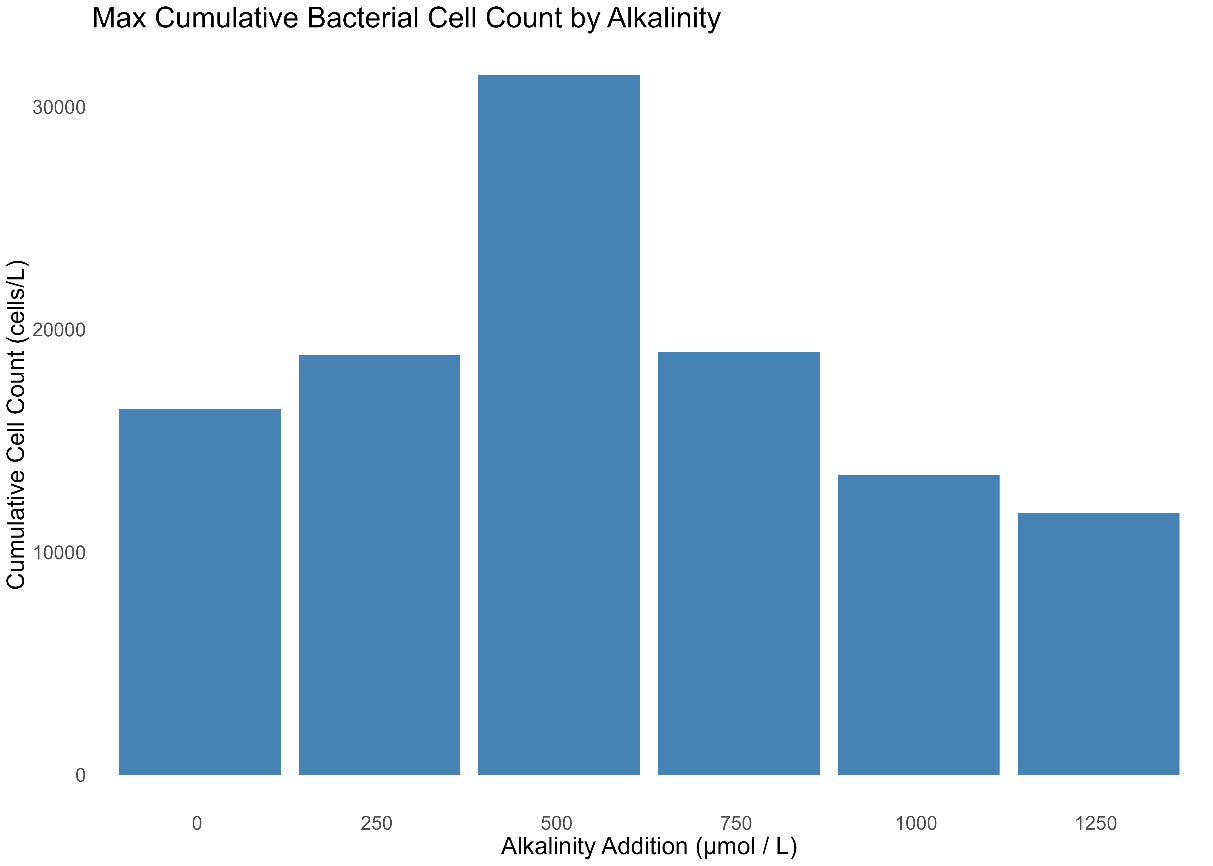


**Supplementary Figure 5:** Cumulative bacterial cell counts across mesocosms grouped by alkalinity treatments. The x-axis shows the added alkalinity concentrations (µmol/L), while the y-axis represents the total accumulated bacterial cell counts (cells/µL).

# Water Chemistry

Plots of alkalinity and pH measurements taken during the experiment are shown here to demonstrate that the treatments tested in our study had a measurable impact on water chemistry. It is important to note that our study focuses specifically on the effects of the alkalinity treatment on the bacterial community in the water of the mesocosms. The publication of additional aspects of the experiment is in the hands of GEOMAR and the investigators overseeing the RETAKE project.


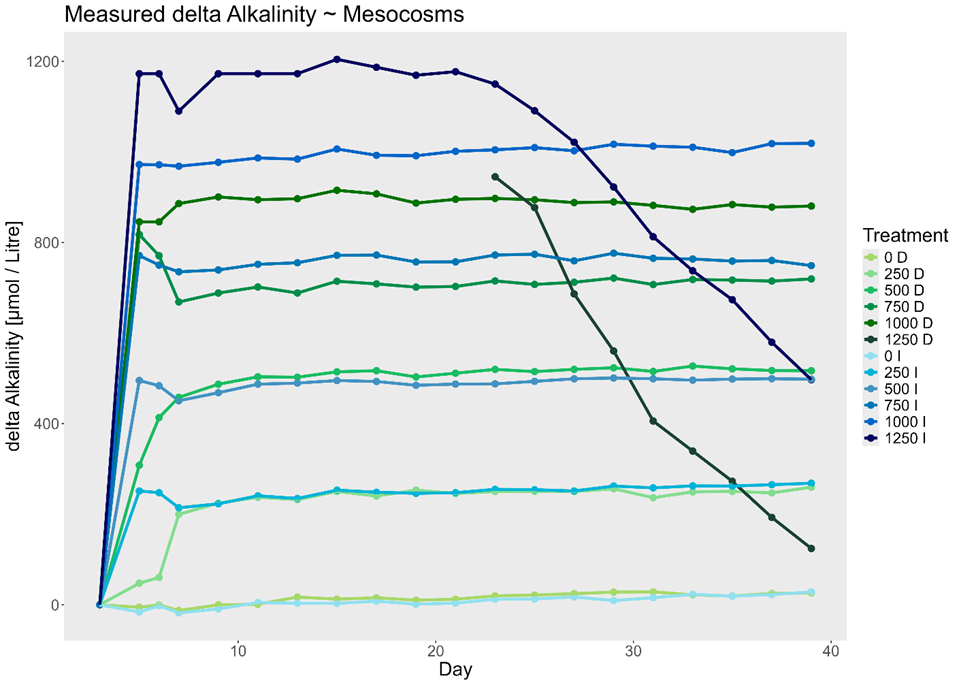


**Supplementary Figure 6.** Delta Alkalinity measured over the course of the experiment in each mesocosm with titration using the Gran method (Gran 1952) and a Metrohm 855 Robotic Titrosampler


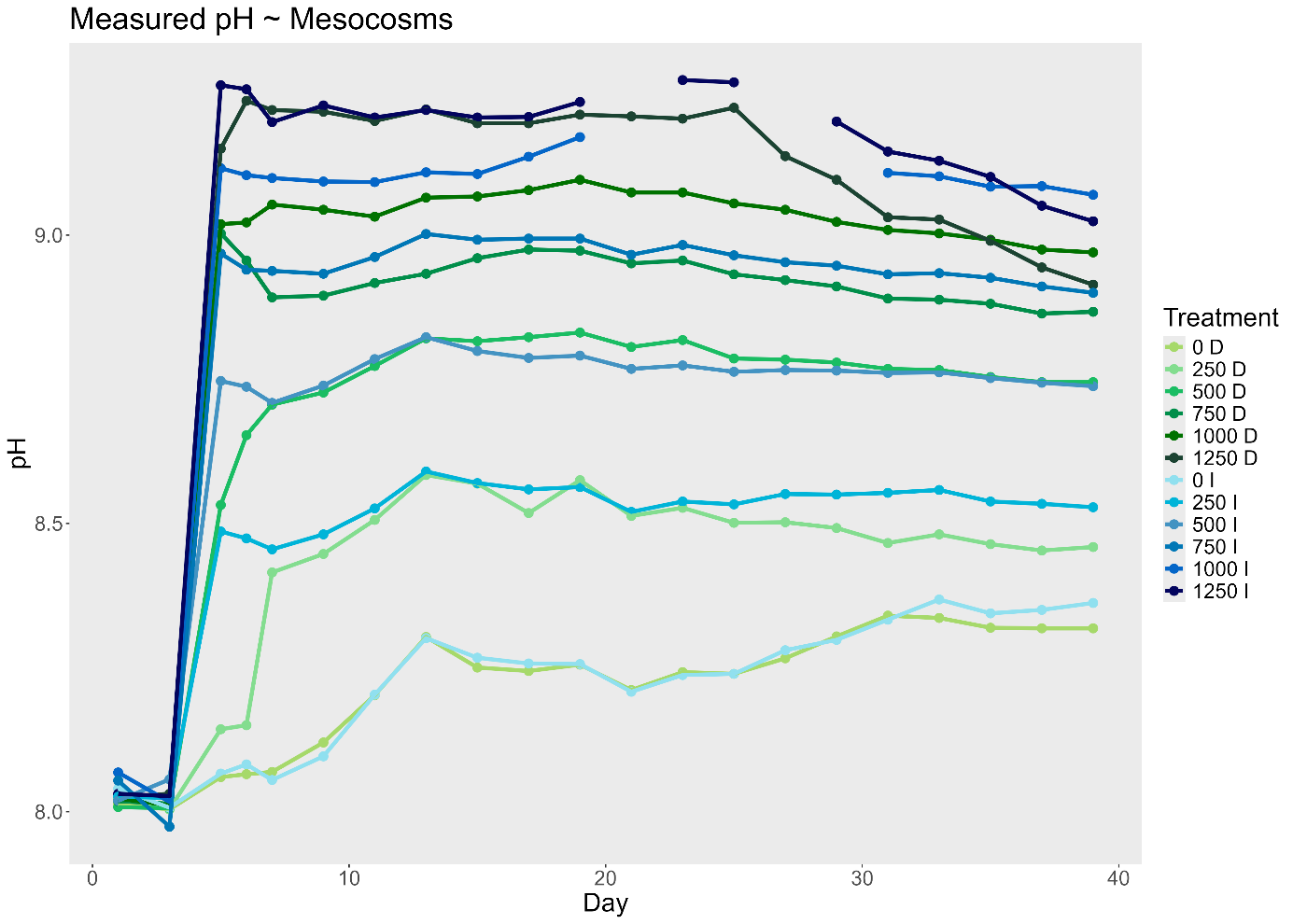


**Supplementary Figure 7.** pH measured over the course of the experiment in each mesocosm. pH was measured at 25°C and calculated at 6°C with the CO2SyS using the following parameters: Lueker 2000, KSO4 of Dickson, pH Total scale, Uppstrom 1974

Supplementary table 1: (PCR specifications)

| Master mix | |  | Cycling Conditions | | |
| --- | --- | --- | --- | --- | --- |
| Component | Amount |  | **Step** | **Temperature** | **Duration** |
| DNA Template | 1-10 ng (1µl) |  | Pre-denaturation | 96°C | 60 s |
| Primer 515F-Y (5′-GTGYCAGCMGCCGCGGTAA) | 15 pmol |  | Denaturation | 96°C | 15 s |
| Primer 926R (5′-CCGYCAATTYMTTTRAGTTT) | 15 pmol |  | Annealing | 55°C | 30 s |
| MyTaq Buffer (1x) | 20 µL |  | Extension | 70°C | 90 s |
| MyTaq DNA Polymerase (Bioline GmbH) | 1.5 units |  | Final Hold | 8°C | Indefinite |
| BioStabII PCR Enhancer (Sigma-Aldrich Co.) | 2 µL |  |  |  |  |
